# A forecasting method of multi-category product sales: analysis and application

**DOI:** 10.1007/s44176-023-00012-9

**Published:** 2023-03-14

**Authors:** Jing Wang, Ling Luo

**Affiliations:** 1 College of Information Engineering, Wuchang Institute of Technology, Wuhan, 430065 Hubei China; 2 School of Management, Wuhan University of Technology, Wuhan, 430070 Hubei China; 3 School of Civil Engineering, Wuhan University, Wuhan, 430072 Hubei China

**Keywords:** Category feature, Multi-category product, Forecasting method, Retail industry

## Abstract

To solve the problems of high prediction costs and difficult practices in multi-category product classification in the retail industry, optimize the inventory, and improve resilience, this work introduces a forecasting method for multi-category product sales. The forecasting method divides the data into a category set and a numerical set, uses the stacking strategy, and combines it with catboost, decision tree, and extreme gradient boosting. During the feature engineering process, the ratio and classification features are added to the category feature set; the sales at *t* are used for training to obtain the prediction at (*t* + *1*); and the features used in the prediction at time (*t* + 1) are generated by the prediction results at* t*. The update processes of the two sets are combined to form a joint feature update mechanism, and multiple features of* k* categories are jointly updated. Using this method, data of all categories of retail stores can be linked so that historical data of different categories of goods can provide support for the prediction of each category of goods and solve the problem of insufficient product data and features. The method is verified on the retail sales data obtained from the Kaggle platform, and the mean absolute error and weighted mean absolute percentage error are adopted to analyze the performance of the model. The results reveal that the forecasting method can provide a useful reference to decision-makers.

## Introduction

Over the past few decades, various industries have adopted digital technologies to strengthen data-based applications. Intelligent forecasting in retail sales based on cheap and accurate machine learning (ML) not only provides more accurate inventory management but also decreases inventory losses and reduces goods detention costs. It also reduces economic losses caused by the shortage of goods during peak sales, thus improving sales controllability. Under the adverse circumstances of macroeconomic slowdown, e-commerce impact, and public health emergencies, accurate sales forecast is an effective way to solve inventory loss, goods retention, and other challenges of a single product based on digital technology. However, a single total number of sales does not provide suitable information for practical applications. This is because sales data with time series characteristics tend to be stable and periodic after summarizing, and the data structure is simple. Consequently, enterprises cannot accurately stock goods based on the total number of sales. Therefore, managers require detailed short-term product forecasting. In addition, it is fundamental in the retail industry to control costs, provide quality services, and assess market competition.

Research on sales forecasting originated from the autoregressive model proposed by Yule (1927). Later, the moving average part was added to the model, and the auto-regressive moving average model was proposed (Box and Jenkins 1970). To solve the non-stationary problem of time-series data, a differential autoregressive moving average model was proposed (Tang et al. 1991; Heydon and Najork 1999). With the development of ML, many learning methods have been employed to complete the learning task, reduce generalization errors, and improve model performance. The integrated learning methods can be roughly classified into bagging, boosting, and stacking methods (Zhang 2003; Kim 2003; Rubio et al. 2011). In recent years, these methods have been widely studied (Lu et al. 2014; Silahtarogu and Donertasli 2015; Islamoglu et al. 2015). In 2017, the Catboost algorithm was proposed by Yandex. This algorithm uses a special technique to deal with the category features, which can effectively avoid over-fitting (Polak 2017). After that, a number of studies have realized the automatic extraction of more complex and useful features by constructing models with multiple hidden layers by using integrated learning to improve the sales prediction of combined models (Amin et al. 2019; Terui and Li 2019). To improve the performance of a single prediction model in sales forecasting, Ye et al. predicted the sales of more than 1000 retail stores using the XGboost algorithm and achieved good prediction results (Ye et al. 2017). Zhang applied the stacking strategy for model combination and adopted the sliding window method for feature construction and fusion (Zhang 2020). The results obtained using this method reveal that the prediction performance of a model that considers feature combination is higher than that of a model that considers only a single feature. In general, research related to sales forecast has mainly focused on addressing the problem of total sales forecasting for a single category. However, research on sales forecasting of multi-category products is insufficient.

Since 2020, the COVID-19 pandemic has further raised the demand for short-term multi-category sales forecasting. However, the computational cost of classification and prediction of multi-category products in the retail industry is high, and the implementation is challenging. First, due to the non-stability of the sales cycle, sales data for specific categories of goods may be distributed at different stages throughout the time series, and data in the early and late sales period can cause errors in multi-category sales forecasting. Second, the time series analysis of all products may lead to the loss of original data characteristics after category segmentation (Zhao and Li 2020). In addition, the data volume in a single category is of small size. Third, in actual sales, categories can have different time series characteristics, and the correlation between two or more categories is usually defined based on experience. It is challenging to create a single model with time series features covering different product categories. However, modeling each category separately results in a low model training efficiency and large waste of characteristic information from the original data. Considering the current demand and practical difficulties realistically, it is very important to perform short-term intelligent forecasting of sales with high accuracy and low computational cost according to the commodity categories.

To solve this problem and provide a useful reference for retail sales forecasting, this study comprehensively combines the hybrid-decision tree, hybrid-XGboost, and hybrid-catboost algorithms and proposes a nonlinear, multi-category, and joint feature updating algorithm that associates all data types in retail stores. The ratio and classification features are added to the category feature set, and a feature updater helps to generate the feature for the next predicting step. As the feature updater can generate the features used in the prediction at time (*t* + 1) by the prediction results at* t*, multiple features of* k* categories are jointly updated. Then, the historical data of different categories of goods can provide support for the prediction of each category of goods, solve the separation problem of product features, and provide a useful reference to decision-makers.

## Multi-category mixed model construction method

### Joint feature update

The data and features jointly determine the prediction effect of a hybrid model. To shorten the training time of a hybrid model and improve the prediction accuracy, data are preprocessed from the aspects of feature extraction, feature selection, and feature construction to ensure that the data features not only correlate with the prediction target but also are free from collinearity. This method divides data into a numerical set ${N}_{k}$ and category set ${C}_{k}$ and adopts the label coding method and one-hot method to clean the data and for normalization. The feature extraction is performed for the abstract characteristics set at a higher level to explore the time sequence of inherent law between the historical and current data using the correlation coefficient for feature selection. Because the sales volume of the product has certain periodicity and seasonality, the moving average method, exponential smoothing method, sliding window, and seasonal method (Zhou and Guo 2020; Wang and Li 2020) are adopted to acquire new features. Then, the feature construction is performed.

The feature set *M* is expressed as follows:

1 $$M=\{{N}_{k},{C}_{k}\}$$

where ${N}_{k}$ represents the numerical features in the sales data of category *k* (e.g., date, price, cost, and other features affecting sales), and ${C}_{k}$ represents the classification features (e.g., holiday, popular region, and consumable property).

There is a certain correlation between ${N}_{k} \mathrm{and }{C}_{k}$ features, and *M* is continuous data prediction, which helps improve the applicability of subsequent modeling.

The date in the time feature is transformed into a datetime format, and the sales category and sales volume are converted to the floating-point format. Samples that do not contain the date and category feature are considered invalid and removed from the training set. In addition, a week in which the sales data are missing is regarded as non-sales, and this feature is treated with a zero-fill method.

For category features, an encoding method is used to convert string data to numerical data. The label coding method is used for the store and department features, which are sorted numerically in ascending order, starting from one. In addition, to make full use of the aggregate help model for continuous data prediction and improve accuracy, the data reflecting the trend of the sales curve are updated with the corresponding numerical characteristics based on the moving average method (De Livera et al. 2011). Further, to obtain the seasonal trend, the seasonal features are processed by quadratic exponential smoothing to extract the seasonal characteristics (Ramos et al. 2015). Based on different proportions of different categories in total sales, a new ratio feature is constructed mainly by calculating the sales objective weights, which range from zero to one; this feature can reflect the overall influence on a part to a certain extent. Data that are far from the current time are given relatively small weight, while recent data are given relatively larger weight. When the ratio feature is added to the model training, the total sales volume provides certain support for the prediction of all types of goods and helps to reduce the noise in the sample data.

For numerical features, the actual and predicted values are used to obtain weekly and monthly mean values of sales. The sliding window method is used because the average and total sales volumes of retail stores have a certain regularity. The sales data of the previous month are used as training data to predict the sales volume of the following week. The prediction at time (*t* + 1) is obtained based on the prediction results at time* t*; features used in the prediction at time (*t* + 1) are also generated by the prediction results at* t*, which poses a great challenge to the effectiveness of the multi-category product sales forecasting analysis method (Wang and Shu 2022).

**Fig. 1:**
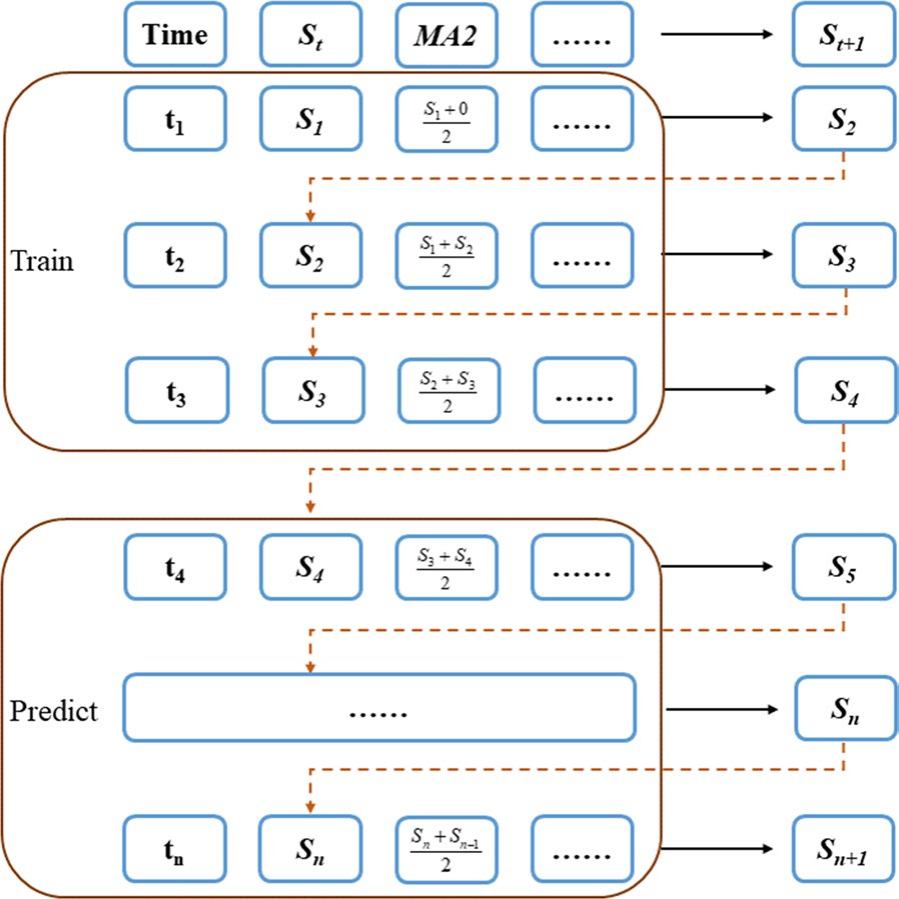
An example of the joint feature update

In this study, the joint feature update uses an iterative prediction method. An example of the method is depicted in Fig. 1. In this example, *S*_*t*_ and *MA2* are two features; *S*_*t*_ represents the sales at time *t*; and *MA2* represents the average value of the sales at times *t* and (*t − 1*). The sales at *t* are used for training to obtain the prediction at (*t* + *1*). Regarding the moving average of two steps, *MA2* at *(t* + *1*) is generated by the average value of sales at time *t* and the predicting sales at (*t* + *1*). Other features are generated by their own equations.

### Hybrid model construction

In sales forecasting, forecasting the future sales based on time series for each category separately has more practical economic value and significance than forecasting the total sales of all categories jointly. Traditionally, the multi-category time series prediction is performed by considering each category separately—a model is developed for each category, and each model uses its own loss function to determine appropriate model parameters, which increases the computational cost significantly. In this study, the integrated characteristics of data are added to the dataset used for model training, and only a single loss function is used to obtain the parameters $\phi$. This reduces the algorithm's computational cost and improves the accuracy of future sales forecasting for multi-category products. The sales data of a commodity are randomly selected from the experimental data and used in the leave-one-out algorithm.

In the proposed multi-category joint feature update prediction optimization algorithm presented, a dataset is expressed as follows:

2 $$D=\{{D}_{k}\}={\left\{\left({{\varvec{x}}}_{kt},{y}_{kt}\right)\right\}}_{t=1..n}$$

where *k* represents the categories based on the time series (e.g., retail store sales dataset); *t* represents time with the length of *n* moments; ${D}_{k}$ represents the sales dataset for category *k*; ${{\varvec{x}}}_{kt}$ represents the data characteristics of the *k*th category at time *t*; ${y}_{kt}$ represents the sales data of the *k*th category at time *t*.

The multi-category hybrid prediction model (Wang and Shu 2022) can be mathematically expressed as follows:

3 $${\widehat{y}}_{kt}=\phi \left({\psi }_{N}\left({x}_{kti}\right):{\psi }_{C}\left({x}_{ktj}\right)\right)$$

where $\left\{t\in {D}_{k},i\in {N}_{k},j\in {C}_{k}, N,C\subseteq M\right\}$, and *N* and* C* are feature categories in feature set *M*; data are updated based on *M*.

The training for $\phi$ in a model comprises the process of finding the parameters in $\phi$ by minimizing the loss function *L*. The loss function (Wang and Shu 2022) adopted in this work is defined as follows:

4 $$\mathcal{L}\left({y}_{kt},{\widehat{y}}_{kt}\right)=\mathcal{L}\left({y}_{kt},\phi \left({\psi }_{N}\left({x}_{kti}\right):{\psi }_{C}\left({x}_{ktj}\right)\right)\right)$$

where ${\widehat{y}}_{kt}$ is the predicted value, and ${y}_{kt}$ is the corresponding actual value.

The single-category ML-based algorithms used in this work include decision tree, XGboost, and catboost. The corresponding multi-category ML-based algorithms are called the hybrid-decision tree, hybrid-XGboost, and hybrid-catboost algorithms. The hybrid algorithms can be used to learn the data of *k* categories in *n* iterations, so there are *k* × *n* data points, so the amount of data is much larger than that of a single model.

### Algorithm parameters

As there are many parameters in the multi-category joint feature update algorithm, each parameter is adjusted separately, and then, the prediction performance of the model is observed on the validation set. However, when this approach is used, the time required for parameter optimization is too long. In the actual forecast, parameter adjustment is usually carried out by experienced engineers and takes a long time. To save labor costs and improve the applicability of the hybrid model, all parameters in this study are set to the default system values without any manual parameter adjustment.

**Fig. 2:**
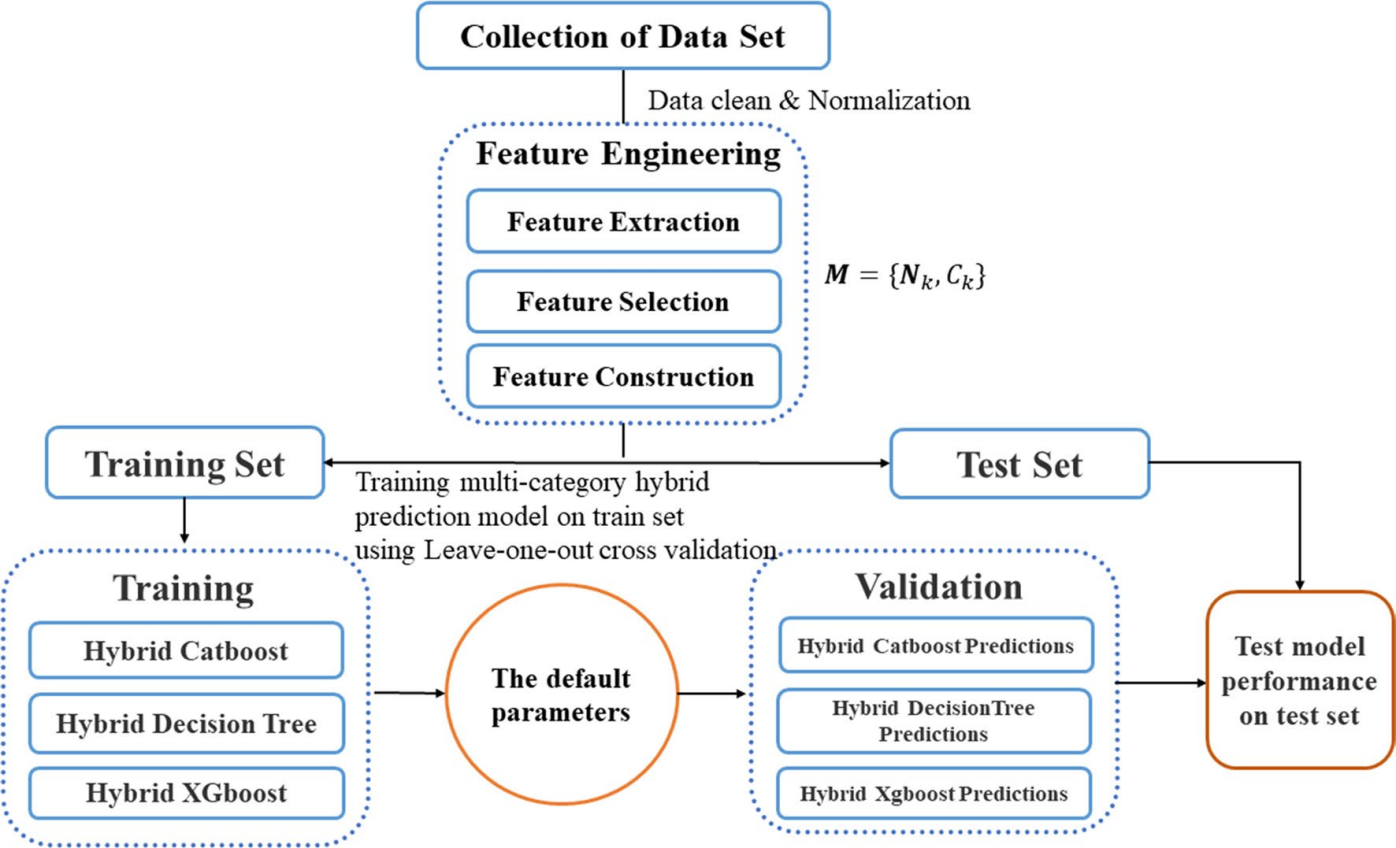
Flowchart of the proposed algorithm

The parameters of the hybrid-catboost algorithm include the following:
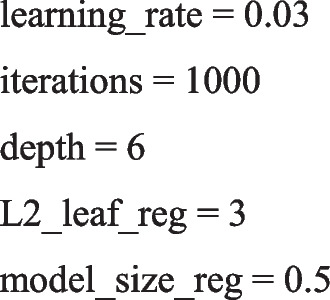


The parameters of the hybrid-decision tree include the following:
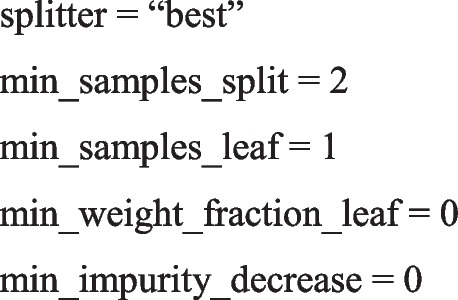


The parameters of the hybrid-XGboost algorithm include the following:
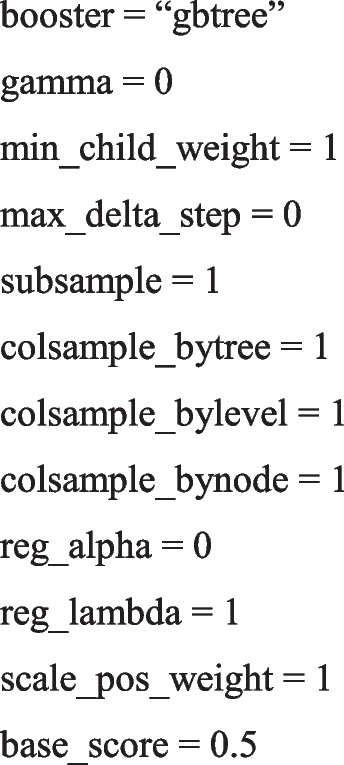


The flowchart of the proposed algorithm is depicted in Fig. 2.

## Empirical analysis and discussion

### Dataset description

The dataset used in this work is the Tv_sales dataset, which is publicly available on Kaggle. This dataset contains daily sales of 84 products from June 1, 2016 to August 31, 2016. A multi-category feature updating algorithm, which combines the hybrid-decision tree, hybrid-XGboost, and hybrid-catboost algorithms, is used to construct a mixed prediction model. The traditional single-category ML-based algorithm and seasonal index smoothing are employed as benchmark methods. Considering the retail sales characteristics, the historical sales data of the past two months are used as a predictive variable of the multi-category product sales forecasting method.

### Comparison and analysis

#### *MAE* evaluation analysis

In this work, the catboost, decision tree, and XGboost models are used to predict the Tv_ sales data by using traditional methods and mixed models. The period of Tv_sales data is three months; the predicted value of *k* for each category is summed monthly (weekly sales added); and the total monthly predicted value ${\widehat{y}}_{kt}$ of each category is determined. Then, the error is obtained by comparing the predicted value with the corresponding true value ${y}_{kt}$ of each category based on the corresponding amount of monthly sum. The mean absolute error (*MAE*) is obtained by summarizing all category errors and dividing the final sum by the total number of categories, which is expressed as follows:

5 $$MAE=\frac{1}{n}\sum_{k=1}^{n}\left|{\widehat{y}}_{kt}\right.-\left.{y}_{kt}\right|$$

where $$\left\{t\in {D}_{k}\right\}$$.

**Fig. 3:**
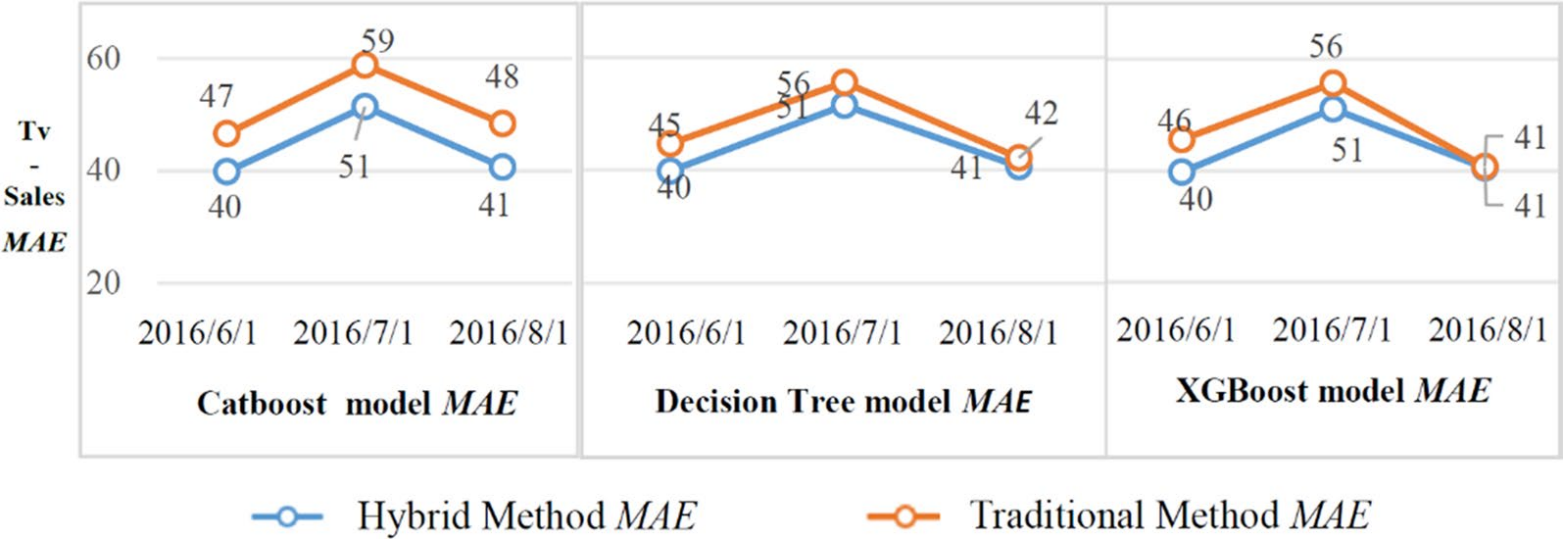
The *MAE* of the forecast using the Tv_Sales dataset

The specific situation is depicted in Fig. 3.

As presented in Fig. 3, the hybrid-catboost, hybrid-decision tree, and hybrid-XGboost models had lower average absolute errors on the multi-category Tv_sales datasets than the traditional single-category models. This indicates that the sales forecasting result of the hybrid multi-category model is more accurate than that of the traditional single-category method.

For time-series data, the same model may not be valid on different datasets. Therefore, it is necessary to test and select appropriate models according to different datasets. This has been a common challenge in the ML field. The comprehensive results indicate that adopting a hybrid-decision tree algorithm can greatly improve the accuracy of multi-category product sales prediction for all datasets. The XGboost model achieved a significant improvement after the adoption of the multi-category method. Generally, the proposed multi-category retail sales forecasting method has a more stable forecasting effect than the single-category method. This is because the proposed method can avoid the phenomenon of ill-conditioned forecasting caused by a small number of data points.

#### *WMAPE* evaluation analysis

It is challenging to compare the *MAE* results to evaluate the improvement effect of the models. Therefore, a weighted mean absolute percentage error *(WMAPE*) is adopted as an evaluation index. It provides a solution to situations where actual sales are zero at *t*. The calculation method is the sum of (predicted value ${\widehat{y}}_{kt}$ − true value ${y}_{kt}$) absolute values divided by the sum of true sales values, and it is mathematically expressed as follows:

6 $$WMAPE = \frac{{\sum\nolimits_{k = 1}^{n} {\left| {\mathop {\hat{y}}\nolimits_{kt} - \mathop y\nolimits_{kt} } \right|} }}{{\sum\nolimits_{k = 1}^{n} {\mathop y\nolimits_{kt} } }}100\%$$

where $$\left\{t\in {D}_{k}\right\}$$.

The *WMAPE* of a single model (catboost, decision tree, and XGboost in this study) is called traditional *WMAPE*. The *WMAPE* of the corresponding multi-category hybrid prediction method is called hybrid *WMAPE*. The error reduction rate in Table 1 is the difference between hybrid *WMAPE* and traditional *WMAPE*. The specific *WMAPE* results are presented in Table 1.

**Table 1:** The *WMAPE* results of the three methods on the Tv_sales dataset

Model	Datetime	Hybrid *WMAPE*	Traditional *WMAPE*	Error reduction rate
Catboost model	2016/6/1	35.166%	39.050%	3.884%
	2016/7/1	41.644%	45.579%	3.935%
	2016/8/1	47.767%	48.793%	1.026%
	Combined result	40.474%	43.692%	**3.218%**
Decision tree model	2016/6/1	35.216%	39.635%	4.419%
	2016/7/1	41.718%	46.170%	4.452%
	2016/8/1	47.826%	52.604%	4.778%
	Combined result	40.535%	45.036%	**4.501%**
XGBoost model	2016/6/1	35.131%	40.471%	5.340%
	2016/7/1	41.482%	46.467%	4.985%
	2016/8/1	47.913%	51.472%	3.559%
	Combined result	40.440%	45.220%	**4.779%**

By comparing the result of hybrid *WMAPE* and traditional *WMAPE* data, the error rate of the three models is reduced. After the catboost model used a multi-category hybrid prediction method, its *WMAPE* decreased by an average of 3.218% over the three months. The *WMAPE* of the mixed multi-category decision tree prediction model is reduced by 4.501% in three months. The error reduction rate of the XGboost regression prediction algorithm is reduced by 4.779% in three months. The results indicated that on the Tv_sales dataset of the retail industry, the prediction performance of the multi-category combined feature update hybrid prediction method performed better than the three traditional methods.

Among the three multi-category joint feature hybrid algorithms, the decision tree and XGboost regression algorithms showed significant improvement, while the catboost algorithm achieved only slight improvement. As the weighted average percentage error of prediction of the catboost algorithm is the lowest among the three models, it can be concluded that the catboost has a good performance. Catboost uses combinatorial category features to leverage relationships between features, thereby greatly enriching the feature dimension. It can improve the accuracy rate even when it is very close to the maximum accuracy. This indicates that using the hybrid method can help the model determine the nature of the relationship between the time-series data of different categories of products. Generally, the multi-category product sales forecasting prediction method based on joint feature update achieved better prediction accuracy than the traditional single-category method, so it can provide more detailed data support to decision-makers.

### Discussion

Accurate category sales forecasting has always been an important part of the retail industry, especially in the context of attaining normalization during the pandemic. The proposed hybrid model can effectively improve the rate of stocking, saving costs, and reducing inventory wastage. According to the empirical analysis results, the multi-category sales forecast of the retail industry can be optimized in the two following aspects: digital age retail enterprises can combine a variety of factors influencing sales, which can make the positive category sales forecast more accurately; increase the ratio feature, making it more scientific and reasonable by using historical data mining information; provide better data samples and data relationship characteristics; improve the accuracy of a sales forecasting model; and provide an effective path for retail inventory optimization.

The proposed hybrid sales forecasting method based on joint feature updating has higher accuracy and smaller error than the traditional methods. The proposed method can be used for multi-category sales forecasting in the retail industry to balance the contradictions between the efficiency and accuracy of the forecasting model effectively, providing valuable decision-making information to decision-makers.

## Conclusions

Compared with traditional methods, the multi-category forecasting method can solve the problem of insufficient product data and features. The ratio and classification features are added to the category feature set, and the historical data of different categories of goods can provide support for the prediction of each category of goods. Compared with *k* models that are required for *k* categories, the proposed hybrid algorithm has a better simulation effect, greatly saves computing and storage resources, and has a faster training speed.

Based on the *MAE* and *WMAPE* analyses, the prediction results of multi-category mixed sales obtained by the fusion of multiple algorithms are closer to the real data than the result of a single model. The *MAE* results of the proposed method are lower than those of the traditional model, and the *WMAPE* has a lower error rate. The proposed hybrid method can achieve an average performance improvement of approximately 4% compared with the three traditional methods. Further, compared with a single model, the multi-category sales forecasting method processing is performed based on the total number of products and the correlation between features and category attributes, and multiple features of* k* categories are jointly updated. The multi-category hybrid model uses default parameters to improve the training speed and save computing and storage resources. Compared with the existing methods, the proposed method has a lower computational cost and a better model-fitting effect.

Effective multi-category sales forecasting can provide good data support to the retail industry, which can appropriately devise the inventory plan and improve the anti-risk ability. The proposed hybrid forecasting method based on joint feature updating can not only serve as a reference in the retail industry but can also be applied to other similar sales forecasting industries.

## Data Availability

Data are available on reasonable request.

## Abbreviations

ML: Machine learning
Catboost: Gradient boosting + categorical features
XGboost: EXtreme gradient boosting
*MA2*: The average value of the sales at times *t* and (*t **−** 1*)
*MAE*: Mean absolute error
*WMAPE*: Weighted mean absolute percentage error
